# SWATH Mass Spectrometry-Based CSF Proteome Profile of *GBA*-Linked Parkinson’s Disease Patients

**DOI:** 10.3390/ijms232214166

**Published:** 2022-11-16

**Authors:** Saima Zafar, Aneeqa Noor, Neelam Younas, Mohsin Shafiq, Matthias Schmitz, Isabel Wurster, Kathrin Brockmann, Thomas Gasser, Inga Zerr

**Affiliations:** 1 Clinical Department of Neurology, University Medical Center Göttingen and the German Center for Neurodegenerative Diseases (DZNE), Robert-Koch-Straße 40, 37075 Göttingen, Germany; 2 Biomedical Engineering and Sciences Department, School of Mechanical and Manufacturing Engineering (SMME), National University of Sciences and Technology (NUST), Bolan Road, H-12, Islamabad 44000, Pakistan; 3 Institute of Neuropathology, University Medical Center Hamburg-Eppendorf, Martinistraße 52, 20246 Hamburg, Germany; 4 Department of Neurodegenerative Diseases, Hertie-Institute for Clinical Brain Research, University of Tübingen, Hoppe-Seyler-Str. 3, 72076 Tübingen, Germany; 5 German Center for Neurodegenerative Disease (DZNE), 72076 Tübingen, Germany

**Keywords:** β-glucocerebrosidase, proteomics, CSF, SWATH, α-synuclein

## Abstract

β-glucocerebrosidase (*GBA*)-associated mutations are a significant risk factor for Parkinson’s disease (PD) that aggravate the disease pathology by upregulating the deposition of α-Synuclein (α-Syn). The resultant clinical profile varies for PD patients without *GBA* mutations. The current study aimed to identify the proteomic targets involved in the pathogenic pathways leading to the differential clinical presentation of *GBA*-associated PD. CSF samples (*n* = 32) were obtained from PD patients with *GBA* mutations (*n* = 22), PD patients without *GBA* mutations (*n* = 7), and healthy controls that were carriers of *GBA* mutations (*n* = 3). All samples were subjected to in-gel tryptic digestion followed by the construction of the spectral library and quantitative SWATH-based analysis. CSF α-Syn levels were reduced in both PD_Idiopathic_ and PD_GBA_ cases. Our SWATH-based mass spectrometric analysis detected 363 proteins involved in immune response, stress response, and cell signaling in various groups. Intergroup analysis showed that 52 proteins were significantly up- or downregulated in various groups. Of these 52 targets, 20 proteins were significantly altered in PD_GBA_ cases only while 2 showed different levels in PD_Idiopathic_ patients. Our results show that the levels of several pathologically relevant proteins, including Contactin-1, Selenium-binding protein 1, Adhesion G Protein-Coupled Receptor, and Apolipoprotein E are significantly different among the sporadic and genetic variants of PD and hint at aggravated synaptic damage, oxidative stress, neuronal loss, and aggregation of α-Syn in PD_GBA_ cases.

## 1. Introduction

Parkinson’s disease (PD) is the second most prevalent neurodegenerative disorder and affects around 1% of the population above the age of 60 [1]. Characterized by the intraneuronal accumulation of α-Synuclein (α-Syn) and extensive death of dopaminergic neurons in the substantia nigra, PD results in debilitating motor symptoms [2,3]. The patients present resting tremor, rigidity, bradykinesia, and postural instability along with multiple non-motor symptoms such as hyposmia, REM sleep behavior disorder, mood swings, and cognitive dysfunction [4,5]. Decades of research have identified ageing, environmental toxicants (e.g., pesticides and herbicides), and genetic predisposition as the major risk factors for this disorder [6,7,8].

Although genome-wide association studies have identified >80 risk loci for PD, mutations in β-glucocerebrosidase (*GBA*) are amongst the most significant risk factors [9,10]. Mutations in the *GBA* gene were initially discovered in association with Gaucher’s disease whereby a decrease in the activity of *GBA* leads to abnormalities in the metabolism of glucosylceramide and multisystem damage [11]. Over the last decade, an increasing amount of evidence has correlated the occurrence of PD with various *GBA* mutations. Although around 300 mutations have been reported for the *GBA* gene, not all play an equal role in the pathogenesis of PD [12]. L444P, N370S, and E326K mutations are most common among PD patients and, interestingly, the occurrence of E326K exclusively results in PD and has no role in Gaucher’s disease [13]. Furthermore, these PD-associated mutations show differences in PD risk, biological profiles, and clinical trajectories based on mutation severity.

*GBA* mutations have various effects on the pathophysiology of PD [14,15]. Loss-of-function mutations in the *GBA* gene have been hypothesized to increase the accumulation of α-Syn, possibly initiating and aggravating the neurodegenerative process in PD [16]. PD patients with *GBA* mutations, compared with those without mutations, have been reported to present a characteristic biological and clinical phenotype with reference to aggregation of α-Syn, impaired lipid metabolism, cognitive dysfunction, disease progression, and survival time, indicating a definite effect of *GBA* mutations on the prognosis of PD [16,17,18,19,20,21,22]. Although the role of the *GBA* gene has been established in PD, we lack proteome-wide studies in human subjects that may allow us to pinpoint the factors that are being affected by these mutations and establish an understanding of the disorder.

Cerebrospinal fluid (CSF) is being extensively used to understand the pathophysiology of neurodegenerative disorders and hunt for potential biomarkers. In contrast to other biological fluids, CSF has the advantage of providing a direct window into disease-associated changes in the nervous system. Previous studies have reported the relative levels of key pathogenic proteins in CSF from PD patients with and without *GBA* mutation to highlight the differences in the pathophysiology [20]. However, the lack of proteome-wide studies has limited our understanding of the CSF signature of PD proteome to very few targets. With the recent advances in mass spectrometry (MS), it is now possible to accurately identify and quantify proteins in biofluids. Approaches such as sequential window acquisition of all theoretical spectra (SWATH) provide an ideal platform to analyze complex protein samples reproducibly and predict potential biomarkers confidently [23].

In the current study, we used the advantages of SWATH to analyze and quantify globe-wide proteome alterations in CSF samples from PD patients with and without *GBA* mutations and identify the differentially regulated pathological targets to decipher the role this mutation in PD.

## 2. Results

### 2.1. GBA-Mutation-Linked Proteomic Alterations in PD Patients

**Figure 1:**
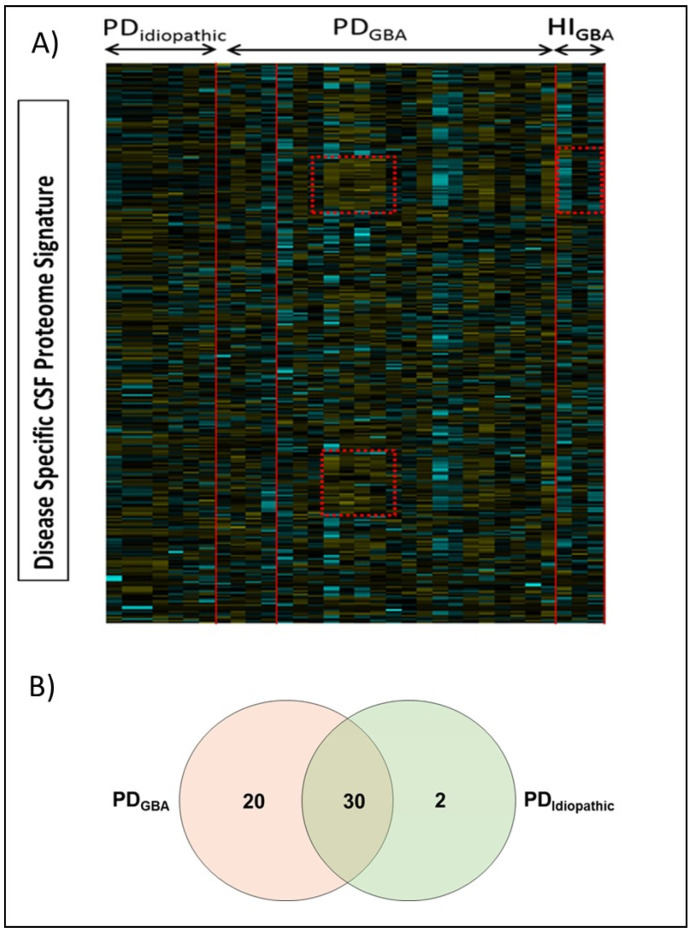
Disease-specific alterations in CSF proteome. (**A**) The heat map shows the relative levels of identified proteins across all analyzed samples. The red lines mark clustering in response to *GBA* genotype in PD cases. Both rows and columns were clustered using correlation distance and average linkage and these clusters are represented by red lines (**B**) Venn diagram presenting the number and distribution of differentially regulated proteins of each group (in comparison to controls).

We created a spectral library with a traditional data-dependent acquisition (DDA) mass spectrometry method. It contained approximately 255 distinct protein groups and 2854 peptides, and 81,037 spectral counts were identified with greater than 99% confidence and passed the false discovery rate (FDR) from fit analysis using a critical FDR of 1%. However, at a global 1% FDR, 290 distinct protein groups, 3915 peptides, and 101,293 spectral counts were identified with greater than 99% confidence by using ProteinPilot^TM^ software (Supplementary File S1). Following the generation of the spectral library, the identification and quantification of *GBA*-linked altered proteins and an intergroup analysis was conducted to identify the differentially regulated targets (Supplementary File S2). Overall, we were able to identify altered proteins from the generated spectral libraries and could establish clear differences in amount of the same proteins in response to *GBA* mutation with high confidence (Figure 1A). After removing the common contaminants, 52 differentially regulated proteins were obtained. Of these 52 targets, 20 proteins were significantly altered in PD_GBA_ cases only while 2 showed different levels in PD_Idiopathic_ patients. Additionally, subtype-specific analysis showed the number of uniquely regulated proteins, in comparison to HI in PD cases with and without *GBA* mutations (Figure 1B). 

### 2.2. Pathway Analysis and Functional Characterization

A comprehensive pathway and functional analysis, using GO in conjunction with Panther, Uniprot, and ProteinPilot software, allowed us to annotate CSF proteins to various subgroups based on their functions. Most of the identified proteins were found to play a role in immune response, stress response, and response to an external and biotic stimulus. The components of the Wnt signaling pathway, plasminogen activating cascades, and Cadherin signaling pathways were most abundantly present in the CSF. In accordance with these findings, most of the proteins could be assigned to the class of defense proteins or were enzymes/enzyme modulators. Moreover, most of the proteins detected in CSF were of extracellular origin. Supplementary Figure S1 shows a detailed overview of the functional subclasses and roles of identified proteins along with the number of proteins specific to each subgroup.

### 2.3. Proteomic Alterations in Response to GBA-Mutation in PD Patients

We compared the profiles of CSF proteins of all groups to identify differentially regulated proteins that may play a role in disease pathology and were able to identify 52 significantly altered proteins for various groups. In PD_GBA_, 20 proteins were found to be significantly altered in comparison to HI, but not PD_Idiopathic_ cases (Supplementary Figure S2). In comparison to the other groups, PD_idiopathic_ cases presented two unique proteins, all of which were significantly upregulated (Supplementary Figure S3). The former category contained mainly secreted and membrane-bound proteins involved in neuronal structure (Neurotrimin, Neural cell adhesion molecule, Neural cell adhesion molecule 1, Fibronectin, Receptor-type tyrosine-protein phosphatase delta, Seizure 6-like protein, Fibulin 1, Fibulin 5, and Reticulon-4 receptor), neurotransmission (Isoform 2 of Amyloid-like protein 1), protein folding/ degradation (Clusterin, Plasma protease C1 inhibitor, Antithrombin -III), transport (Alpha-1-acid glycoprotein 2), immunity (Complement C6, Prostaglandin-H2), and metabolism (Galectin-3-binding protein, B4GALT1, Sulfhydryl oxidase 1, Alpha-mannosidase 2×). The two targets in the latter category were involved in metabolism (Beta hexoaminidase) and antioxidation (extracellular superoxide dismutase).

The remaining targets were significantly altered in both variants of PD. Within this category, 12 proteins showed a significant reduction in comparison to HI in both PD_Idiopathic_ and PD_GBA_ cases (Supplementary Figure S4). This category involved secreted, cytoplasmic, and membrane-bound neuronal structure (Contactin-2, Cell surface glycoprotein MUC 18, Gelsolin, Neural cell adhesion molecule 2, Neural cell adhesion molecule L1), neurotransmission (Ephrin type-A receptor 4), protein folding/degradation (Procollagen C-endopeptidase enhancer 1, Alpha-2-macroglobulin), and metabolism (Aspartate aminotransferase, Pyruvate kinase, L-lactate dehydrogenase, Transcobalamin-2).

**Figure 2:**
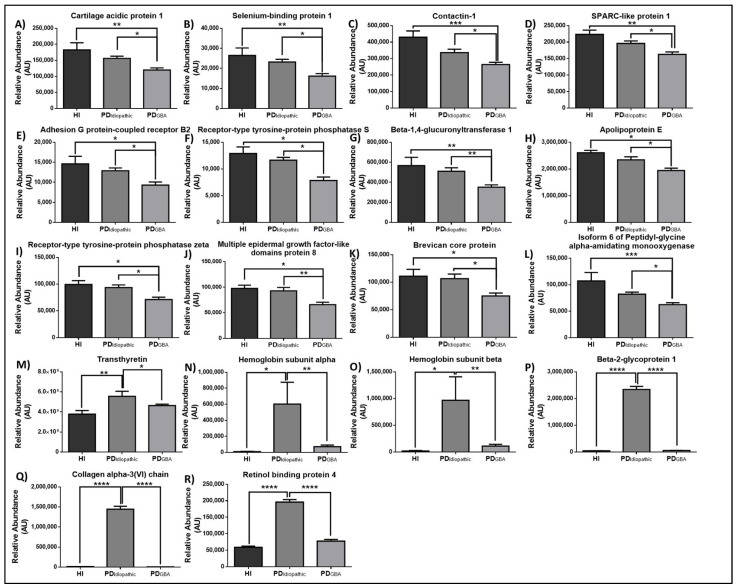
Relative CSF concentrations of the proteins differentially regulated in PD_Idiopathic_ vs PD_GBA_ cases. The graphs depict the relative amount of (**A**) Cartilage acidic protein 1, (**B**) Selenium-binding protein 1, (**C**) Contactin 1, (**D**) SPARC-like protein 1, (**E**) Adhesion G Protein-Coupled Receptor B2, (**F**) Receptor-type tyrosine-protein phosphatase S, (**G**) Beta-1,4-glucuronyltransferase 1, (**H**) Apolipoprotein E, (**I**) Receptor-type tyrosine-protein phosphatase zeta, (**J**) Multiple epidermal growth factor-like domains protein 8, (**K**) Brevican core protein, (**L**) Isoform 6 of Peptidyl-glycine alpha-amidating monooxygenase, (**M**) Transthyretin, (**N**) Hemoglobin subunit alpha, (**O**) Hemoglobin subunit alpha, (**P**) Beta-2-glycoprotein 1, (**Q**) Collagen alpha-3(VI) chain and (**R**) Retinol binding protein 4 in HI, PD_Idiopathic_, and PD_GBA_ cases. One-way ANOVA, followed by Tukey’s multiple comparisons test, was used for statistical analysis. Error bars represent SEM. (* *p* ≤ 0.05; ** *p* ≤ 0.01; *** *p* ≤ 0.001; **** *p* ≤ 0.0001).

Significant and clinically relevant differences were evident in PD_Idiopathic_ and PD_GBA_ cases among 18 cytoplasmic and secreted targets involved in neuronal structure (Contactin 1, Receptor-type tyrosine-protein phosphatase S, Receptor-type tyrosine-protein phosphatase zeta, Brevican core protein, Collagen alpha-3(VI) chain), signaling (Cartilage acidic protein 1, SPARC-like protein 1, Adhesion G Protein-Coupled Receptor B2), transport (Apolipoprotein E, Hemoglobin subunit alpha, Hemoglobin subunit alpha, Transthyretin, Beta-2-glycoprotein 1, Retinol binding protein 4), immunity (Multiple epidermal growth factor-like domains protein and 8), and metabolism (Selenium-binding protein 1, Beta-1,4-glucuronyltransferase 1, Isoform 6 of Peptidyl-glycine alpha-amidating monooxygenase) (Figure 2). 

### 2.4. CSF α-Syn Profiles in PD Patients with GBA Mutations

**Figure 3:**
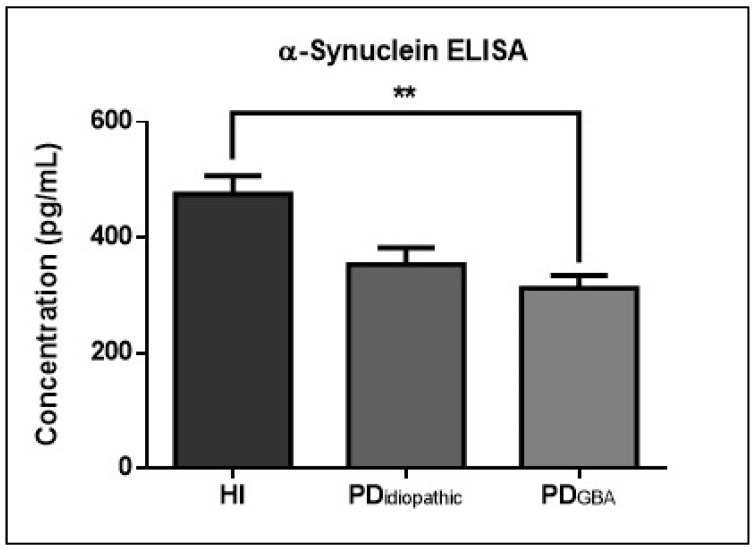
Correlation between α-Syn levels in CSF of PD patients with and without *GBA* mutations. The graph depicts the levels of α-Syn (pg/mL) in CSF from HI, PD_idiopathic_, and PD_GBA_ cases. One-way ANOVA, followed by Tukey’s multiple comparisons test, was used for statistical analysis. Error bars represent SEM. (** *p* ≤ 0.01).

ELISA-based quantification detected *GBA* genotype-associated alterations in CSF α-Syn levels in PD patients (Figure 3). Healthy individuals with *GBA* mutations presented higher levels of α-Syn in comparison to PD patients from both clinical variants; however, this trend was statistically significant in PD_GBA_ cases only. The mean level of α-Syn was slightly higher in PD_Idiopathic_ cases in comparison PD_GBA_ cases. 

### 2.5. Gender-Specific Proteome Alterations in PD Patients with GBA Mutations

**Figure 4:**
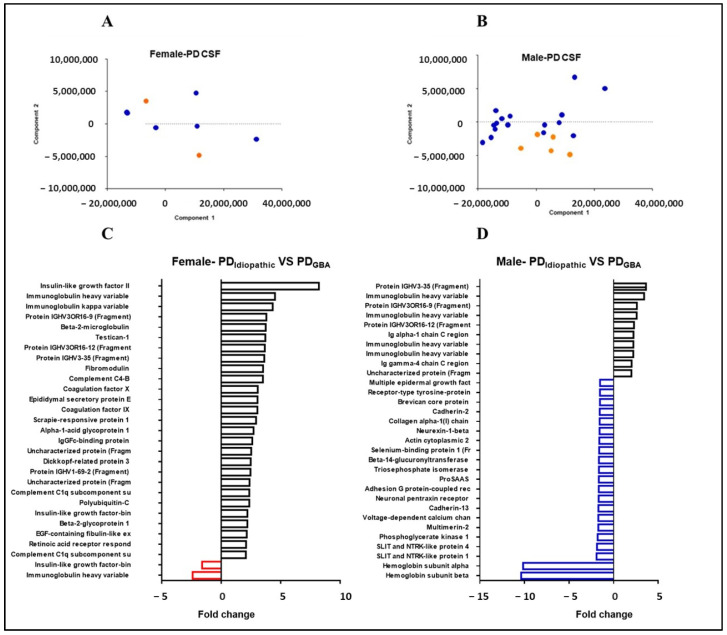
Effect of gender on CSF proteome in PD patients: Principal Component Analysis shows clusters for (**A**) female and (**B**) male cases with (blue circles) and without *GBA* mutation (orange circles). The column bar graph depicts the targets that show two-fold or higher fold change in PD_Idiopathic_ (**C**) female and (**D**) male cases in comparison to the patients carrying a *GBA* mutation. The red and blue columns represent the downregulated proteins in female and male patients respectively.

To elucidate the effects of gender on the CSF proteome of PD patients, we segregated our PD cohort into male and female cases. The PCA plot visualizes the effects of *GBA* mutation on male and female PD patients. Although no obvious clusters can be observed for female cases, possibly due to the low sample number, male samples present clear clustering for samples with and without mutations (Figure 4A,B). We further analyzed the targets in a gender-specific manner to determine the proteins that were accounting for the variation among male and female clusters (Figure 4C,D). Modifications specific to males showed two-fold or more change in 31 proteins while those specific to females depicted changes in 29 targets. However, the latter group had two cases of PD cases without *GBA* mutations so they need to be interpreted with caution.

## 3. Discussion

*GBA* mutations have been previously reported to influence the clinical outcome of PD [24]. Our study analyzed the impact of *GBA* mutations on the CSF proteome in PD patients with the aim of identifying disease triggers and potential biomarkers. For the quantitative assessment of the CSF proteome, we opted for a SWATH MS-based approach that is based on targeted peptide identification using a reference library. It combines the benefits of traditional discovery-based and targeted proteomics resulting in a high-throughput, reproducible, and accurate analysis of proteins in biological samples [25]. In the first study of its kind, we compared the CSF proteome of PD patients with and without *GBA* mutations to healthy controls. Owing to the dynamic and heterogeneous nature of the CSF proteome, it was not surprising that identified proteins belonged to many different functional classes and biological pathways. However, most of our targets were extracellular proteins involved in immune response, neurotransmission, and various signaling pathways. Due to a greater incidence of PD in men, we also targeted the gender-based differences within the proteome of the samples in our cohort [26].

Although all the differentially expressed proteins and their functional annotation provided valuable insights into the pathophysiology of PD, we focused on proteins that were uniquely regulated in certain groups with the aim of identifying possible causes of phenotypic diversity in sporadic and genetic PD. In the first subset, proteomic changes associated with PD_Idiopathic_ cases were targeted. These patients exhibited a significant alteration in the levels of superoxide dismutase whose activity has been previously reported to be reduced in CSF [27]. Similarly, the increased levels of β-hexosaminidase have also been associated with the duration and severity of PD [28]. These proteins, along with the other amyloidogenic (Transthyretin), structural (Collagen), and metabolic (Retinol binding protein 4) proteins hint at the alterations associated with sporadic PD only.

The second subset of altered proteins involved the targets that show significant alterations in PD_GBA_ patients only. This group involved immune (Complement C6, Prostaglandin-H2), transport (Clusterin), signaling (Galectin-3-binding protein, Receptor-type tyrosine-protein phosphatase delta, Alpha-1-acid glycoprotein 2), metabolic (B4GALT1, Plasma protease C1 inhibitor, Sulfhydryl oxidase 1, Antithrombin-III, Isoform 2 of Amyloid-like protein 1), and structural (Neurotrimin, Fibronectin, Seizure 6-like protein, Neural cell adhesion molecule, Neural cell adhesion molecule 1, Reticulon-4 receptor, Fibulin 1) proteins. Interestingly, the trends in this group mimicked the trends observed in α- Syn ELISA hinting at their direct involvement in the pathology of PD. Prostaglandins have been previously reported to modulate the unfolding of amyloids generated in PD and their reduced levels may cause increased aggregation of α-Syn [29]. Reduced levels of proteins involved in cell adhesion have been associated with aberrations in the structure of neurites and altering the ratio of excitatory neurons in comparison with inhibitory neurons, thereby contributing towards neurological disorders [30]. Fibronectin, also downregulated in all PD cases, has been known to exert neuroprotective effects in PD [31].

Proteins that were significantly altered in both PD_Idiopathic_ and PD_GBA_ cases allowed the identification of shared pathological mechanisms. Reduced levels of Gelsolin in both clinical variants can be attributed to its involvement in the aggregation of α-Syn and colocalization with the Lewy bodies [32]. The Neural cell adhesion molecule is also downregulated in all PD cases and is involved in the trafficking and internalization of dopamine [33]. Polymorphisms in Alpha-2-macroglobulin are a risk factor for PD and its CSF levels are also significantly downregulated in all the PD cases targeted in the current study [34].

The most promising targets were identified in the last subset where PD_GBA_ cases differed significantly from PD_Idiopathic_ and HI cases. Reduction in the CSF levels of Contactin-1, a target identified in this subset, has been reported previously and has been associated with synaptic degradation caused by the accumulation of Lewy bodies [35]. In the current study, its levels are significantly lower in PD_GBA_ cases in comparison to PD_Idiopathic_ samples indicating increased synaptic damage. Selenium-binding protein 1 may contribute towards aggravated pathology in PD_GBA_ cases by modulating oxidative stress [36]. The reduced level of Adhesion G Protein-Coupled Receptor has been correlated with dopaminergic cell death and unveils another mechanism which may lead to aggravated pathology in PDGBA patients [37]. Decreased levels of Apolipoprotein E in CSF can be explained by increased aggregation of α-Syn in the brain as the former is known to increase the aggregation of the latter which may influence its release into the CSF [38]. The amount of Hemoglobin, another target identified in this subset, has been known to correlate with the severity of PD [39]. Interestingly, several targets identified in this subset showed a greater fold change in samples from males (known to have a greater incidence of PD) further validating their contribution to the disease pathology.

To establish the impact of *GBA* mutation on α-Syn accumulation, one of the key pathological markers of PD, we compared the levels of α-Syn in CSF. In accordance with the previously published data, our cohort also showed lowered α-Syn levels in CSF from PD patients, possibly in response to its increased aggregation in the brain and decreased elimination into CSF [40,41]. Previous studies have postulated that different *GBA* mutations may have a different impact on the phenotype of PD and these mutations may further reduce the levels of α-Syn in CSF [42,43,44]. However, this difference was not as evident in our cohort, possibly due to the smaller size of the PD_Idiopathic_ group. The mean level of α-Syn was slightly higher in PD_Idiopathic_ cases in comparison to PD_GBA_ cases, but the difference was not significant.

Although *GBA* mutations have been known to increase the incidence of PD and contribute towards its clinical presentation (published previously for the current cohort [45,46]), the exact mechanism of their pathogenicity was not understood. We addressed this problem using proteomic approaches and could highlight some major differences that occur in PD patients in the response to *GBA* mutations. Our results show that the levels of several pathologically relevant proteins, including Contactin-1, Selenium-binding protein 1, Adhesion G Protein-Coupled Receptor, and Apolipoprotein E are significantly different among the sporadic and genetic variants of PD and hint at aggravated synaptic damage, oxidative stress, neuronal loss, and aggregation of α-Syn in PD_GBA_ cases. However, the limited number of samples, especially those of controls (*n* = 3), and the dynamic nature of the CSF proteome demand the replication of the results in a larger cohort. Similarly, the gender-based differences also need to be interpreted cautiously due to a smaller number of female patients and a considerable difference in group size. A comprehensive analysis, that includes sequenced *GBA* mutations in larger cohorts, might be needed to establish the relative pathogenicity of specific polymorphisms on the *GBA* gene in PD in addition to their effects on the age at onset in different genders and their response to various medications. Nevertheless, the outcomes of this study provide important candidates for further validation as diagnostic, prognostic, and therapeutic markers.

## 4. Materials and Methods

### 4.1. Participants and Ethical Approval

A total of 32 participants (Table 1) were included in this study. A detailed neurological assessment was conducted and the patients who fulfilled Movement Disorder Society Clinical Diagnostic Criteria for PD were included in the study. Genetic assessment of *GBA* mutations (N370S, L444P, and E326K) was conducted through genotyping and restriction digestion using slight modifications in previously established criteria [45,46]. The control cases underwent clinical examination to exclude any neurological disorders that may affect the study. All participants provided informed consent prior to the collection of CSF samples and the study was approved by the ethical committee of the University of Tübingen (702/2013BO1).

### 4.2. Sample Collection, Preparation and In-Gel Tryptic Digestion

CSF samples were collected through a lumbar puncture, centrifuged at 2000× *g* for 10 min at 4 °C and stored at −80 °C until further analysis. Samples (50 µL) were concentrated using SpeedVac (sc100, American laboratory trading, Groton, CT, USA) and resuspended in 40 µL of lysis buffer (7M Urea, 2M thiourea and 4% CHAPS, protease, and phosphatase inhibitor cocktail). They were loaded onto a 4–12% NuPAGE Novex Bis-Tris Minigels (Invitrogen, Carlsbad, CA, USA) and allowed to run to a length of 1.5 cm. The gels were subjected to Coomassie staining and protein bands were excised.

In-gel tryptic digestion was performed as previously described [47]. Briefly, the protein bands were diced, reduced, and alkylated followed by overnight digestion with trypsin. The solution containing Tryptic peptides was dried in a SpeedVac and stored at −20 °C until further analysis.

### 4.3. Quantitative SWATH Analysis

For the generation of spectral library, equal amounts of proteins from each sample were pooled to a final concentration of 80 µg and separated into eight fractions using reversed-phase spin columns (Pierce High pH Reversed-Phase Peptide Fractionation Kit, Thermo Fisher Scientific, Waltham, MA, USA). Each fraction was divided into two technical replicates prior to analysis. Digested peptides were dissolved in a loading buffer (2% acetonitrile, 0.1% formic acid in water) to a final concentration of 0.3 µg/µL. For each analysis, 1.5 µg of the analyte was enriched on a pre-column (0.18 mm ID × 20 mm, Symmetry C18-AQ 5 µm, Waters, Milford, MA, USA) and separated on an analytical RP-C18 column (0.075 mm ID × 250 mm, HSS T3, 1.8 µm, Waters). A Nanoflow chromatography system (Eksigent nanoLC425) hyphenated to a hybrid triple quadrupole-TOF mass spectrometer (TripleTOF 5600+) equipped with a Nanospray III ion source (Ionspray Voltage 2400 V, Interface Heater Temperature 150 °C, Sheath Gas Setting 12) and controlled by Analyst TF 1.7.1 software build 1163 (all AB Sciex, Redwood City, CA, USA) was used for analysis.

For the analysis of samples, MS/MS data were acquired using 65 variable size windows across the 400–1050 *m*/*z* range. PeakView Software version 2.1 build 11,041 (AB Sciex, Redwood City, CA, USA) and SWATH quantitation microApp version 2.0 build 2003 were used for generating spectral library and extracting SWATH peaks.

### 4.4. Protein Annotation and Functional Characterization

ProteinPilot Software version 5.0 build 4769 (AB Sciex, Redwood City, CA, USA) was used for protein identification. MS/MS spectra from the combined qualitative analyses were searched against the UniProtKB *Homo sapiens* reference proteome (revision 02-2017, 92,928 entries) augmented with a set of 51 known common laboratory contaminants to identify 363 proteins at a False Discovery Rate (FDR) of 1%. Functional characterization was also achieved through UniProtKB *Homo sapiens* reference proteome and Panther classification system [48].

### 4.5. ELISA

The commercially available ELISA kit (Biolegend, San Diego, CA, USA, catalogue # 844101) was used for the analysis of CSF levels of α-Syn according to the manufacturer’s protocol.

### 4.6. Statistical Analysis

Data visualization and statistical analysis were performed using R. Studio (version 1.1.383) and ClustVis [49]. ANOVA in combination with Tukey Multiple comparisons test were used to identify the differentially regulated targets and *p*-value < 0.05 was considered significant.

## Figures and Tables

**Table 1:** List of samples used in the study. A total of 32 CSF samples were used for the study including 22 PD cases with *GBA* mutation (PD_GBA_), 7 PD cases without mutations (PD_Idiopathic_), and 3 healthy controls with *GBA* mutation (HI_GBA_).

No.	Patient ID	Age	Gender	Age at Onset
PD_Idiopathic_ (Mean age 63.4 ± 7.78 years)				
1	PD_Idiopathic_-1	66	Male	63
2	PD_Idiopathic_-2	74	Male	62
3	PD_Idiopathic_-3	62	Female	53
4	PD_Idiopathic_-4	53	Male	42
5	PD_Idiopathic_-5	60	Male	51
6	PD_Idiopathic_-6	74	Female	67
7	PD_Idiopathic_-7	55	Male	50
PD_GBA_ (Mean age 60.5 ± 9.97 years)				
8	PD_GBA_-1	76	Female	47
9	PD_GBA_-2	58	Male	50
10	PD_GBA_-3	65	Female	55
11	PD_GBA_-4	61	Male	45
12	PD_GBA_-5	44	Male	28
13	PD_GBA_-6	60	Male	49
14	PD_GBA_-7	59	Male	52
15	PD_GBA_-8	51	Male	41
16	PD_GBA_-9	50	Male	44
17	PD_GBA_-10	75	Male	70
18	PD_GBA_-11	41	Male	39
19	PD_GBA_-12	67	Female	56
20	PD_GBA_-13	73	Female	65
21	PD_GBA_-14	63	Male	58
22	PD_GBA_-15	58	Female	51
23	PD_GBA_-16	70	Male	60
24	PD_GBA_-17	67	Male	63
25	PD_GBA_-18	42	Male	40
26	PD_GBA_-19	64	Female	51
27	PD_GBA_-20	59	Male	51
28	PD_GBA_-21	68	Male	56
29	PD_GBA_-22	62	Male	52
HI_GBA_ (Mean age 52.6 ± 13.76 years)				
30	HI_GBA_-1	72	Female	-
31	HI_GBA_-2	45	Male	-
32	HI_GBA_-3	41	Female	-

## Data Availability

Raw data is included as Supplementary Materials. All raw MS files can be obtained from the corresponding author upon request.

## Supplementary Materials

The following supporting information can be downloaded at: https://www.mdpi.com/article/10.3390/ijms232214166/s1.
