# Supplementary material for: SWATH Mass Spectrometry-Based CSF Proteome Profile of GBA-Linked Parkinson’s Disease Patients
Source: Int J Mol Sci. 2022 Nov 16;23(22):14166. doi: 10.3390/ijms232214166 (PMC9699576; doi:10.3390/ijms232214166)
Supplement: Supplementary file 1 [file ijms-23-14166-s001.zip › Caption.pdf]

Supplementary File S1: Raw data for the proteins identified via SWATH-MS;

Supplementary File S2: Raw quantitative data for the identified proteins.

Supplementary Figure S1: Panther-based functional categorization of identified CSF proteins. The graphs depict (A) pathways and (B) biological process manipulated by identified proteins along with their (C) functional class and (D) cellular origin;

Supplementary Figure S2: Relative CSF concentrations of the proteins differentially regulated in PD_{GBA} cases only. The graphs depict the relative amount of (A) Clusterin, (B) Complement C6, (C) Galectin-3-binding protein, (D) B4GALT1, (E) Neurotrimin, (F) Receptor-type tyrosine-protein phosphatase delta, (G) Alpha-1-acid glycoprotein 2, (H) Plasma protease C1 inhibitor, (I) Fibronectin, (J) Sulfhydryl oxidase 1, (K) Seizure 6-like protein, (L) Neural cell adhesion molecule 1, (M) Prostaglandin-H2, (N) Neural cell adhesion molecule, (O) Fibulin 5, (P) Reticulon-4 receptor, (Q) Fibulin 1, (R) Antithromobin-III, (S) Isoform 2 of Amyloid-like protein 1, and (T) Alpha-mannosidase 2× in HI, PDIdiopathic and PDGBA cases. One-way ANOVA, followed by Tukey's multiple comparisons test, was used for statistical analysis. Error bars represent SEM. (* = $p \leqslant 0.05$; ** = $p \leqslant 0.01$; *** = $p \leqslant 0.001$);

Supplementary Figure S3: Relative CSF concentrations of the proteins differentially regulated in $PD_{Idiopathic}$ cases only. The graphs depict the relative amount of (A) Beta-hexosaminidase subunit beta and (B) Extracellular superoxide dismutase in HI, $PD_{Idiopathic}$ and PD_{GBA} cases. One-way ANOVA, followed by Tukey's multiple comparisons test, was used for statistical analysis. Error bars represent SEM. (* = $p \leqslant 0.05$; ** = $p \leqslant 0.01$; *** = $p \leqslant 0.001$; **** = $p \leqslant 0.0001$);

Supplementary Figure S4: Relative CSF concentrations of the proteins differentially regulated in both $PD_{Idiopathic}$ and PD_{GBA} cases. The graphs depict the relative amount of (A) Procollagen C-endopeptidase enhancer 1, (B) Contactin-2, (C) Cell surface glycoprotein MUC18, (D) Aspartate aminotransferase, (E) Pyruvate kinase, (F) Gelsolin, (G) Neural cell adhesion molecule 2, (H) Ephrin type-A receptor 4, (I) L-Lactate dehydrogenase, (J) Transcobalamin-2, (K) Neural cell adhesion molecule L1-like and (L) Alpha-2-macroglobulin in HI, $PD_{Idiopathic}$ and PD_{GBA} cases. One-way ANOVA, followed by Tukey's multiple comparisons test, was used for statistical analysis. Error bars represent SEM. (* = $p \leqslant 0.05$; ** = $p \leqslant 0.01$; *** = $p \leqslant 0.001$; **** = $p \leqslant 0.0001$).
