# Supplementary figures and images for: SWATH Mass Spectrometry-Based CSF Proteome Profile of GBA-Linked Parkinson’s Disease Patients
Source: Int J Mol Sci. 2022 Nov 16;23(22):14166. doi: 10.3390/ijms232214166 (PMC9699576; doi:10.3390/ijms232214166)

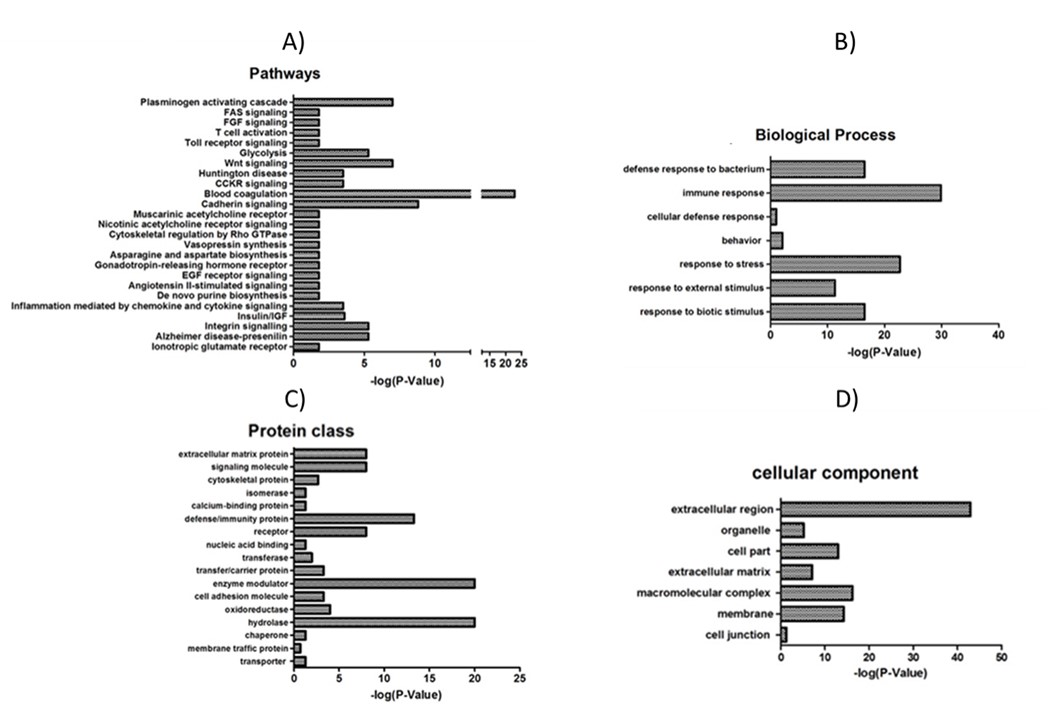

Supplement: Supplementary file 1 [file ijms-23-14166-s001.zip › Supplementary Figure 1.jpg]

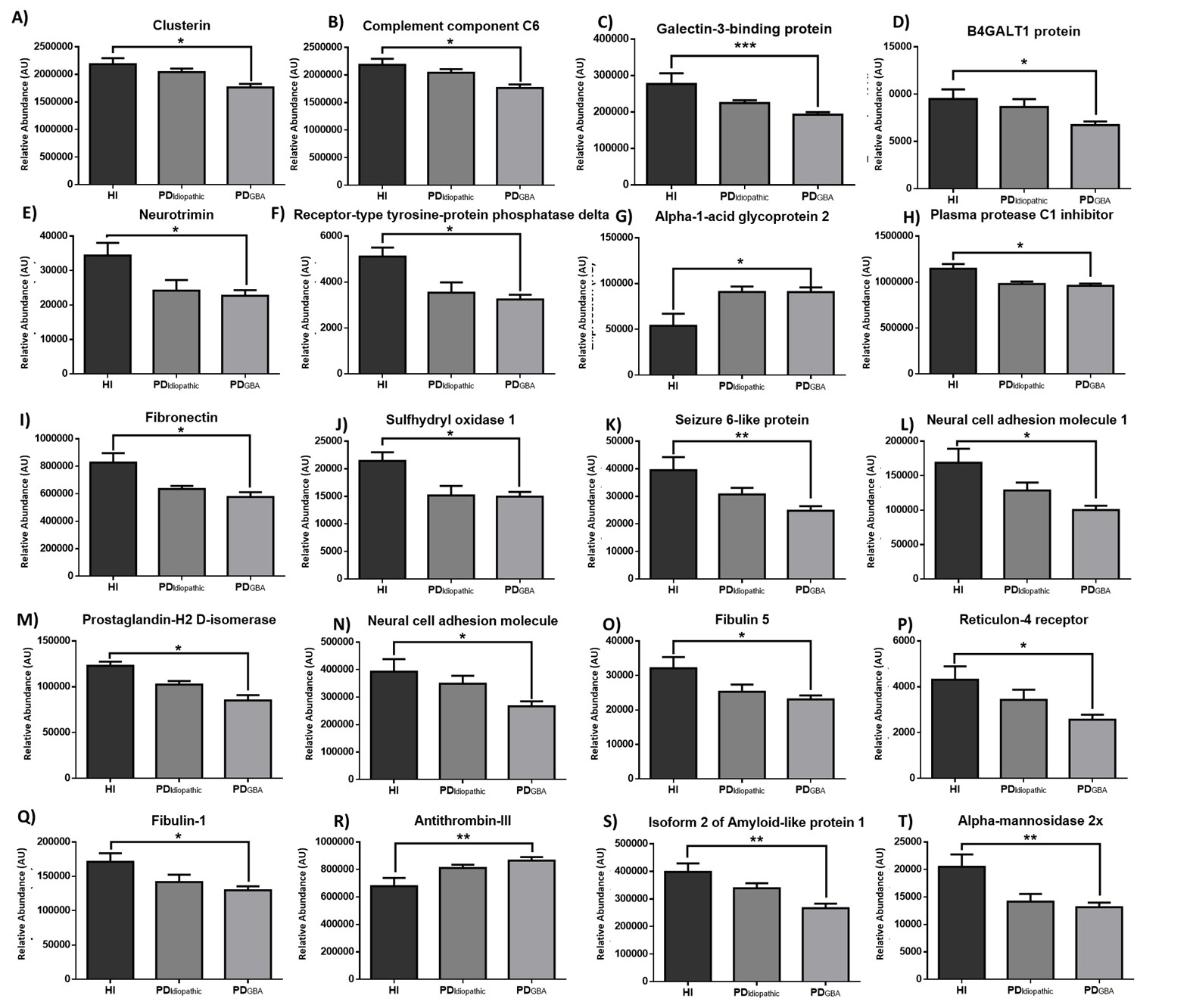

Supplement: Supplementary file 1 [file ijms-23-14166-s001.zip › Supplementary Figure 2.jpg]

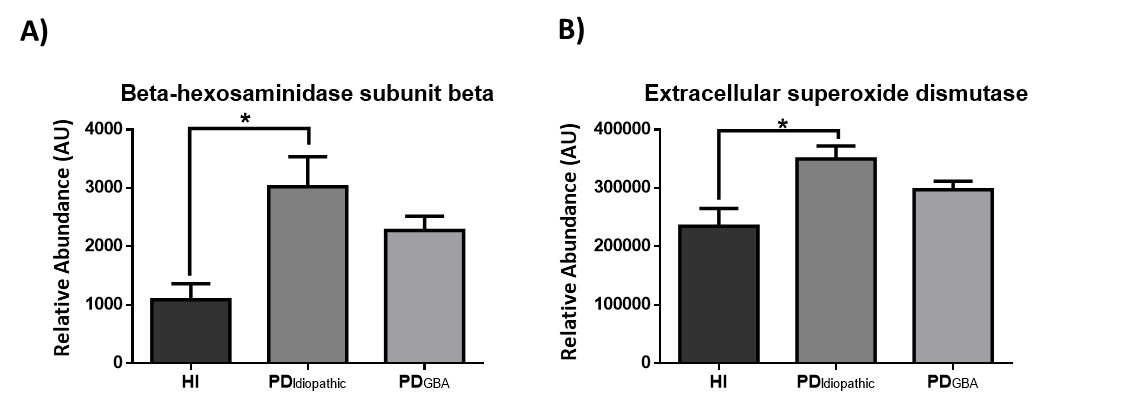

Supplement: Supplementary file 1 [file ijms-23-14166-s001.zip › Supplementary Figure 3.jpg]

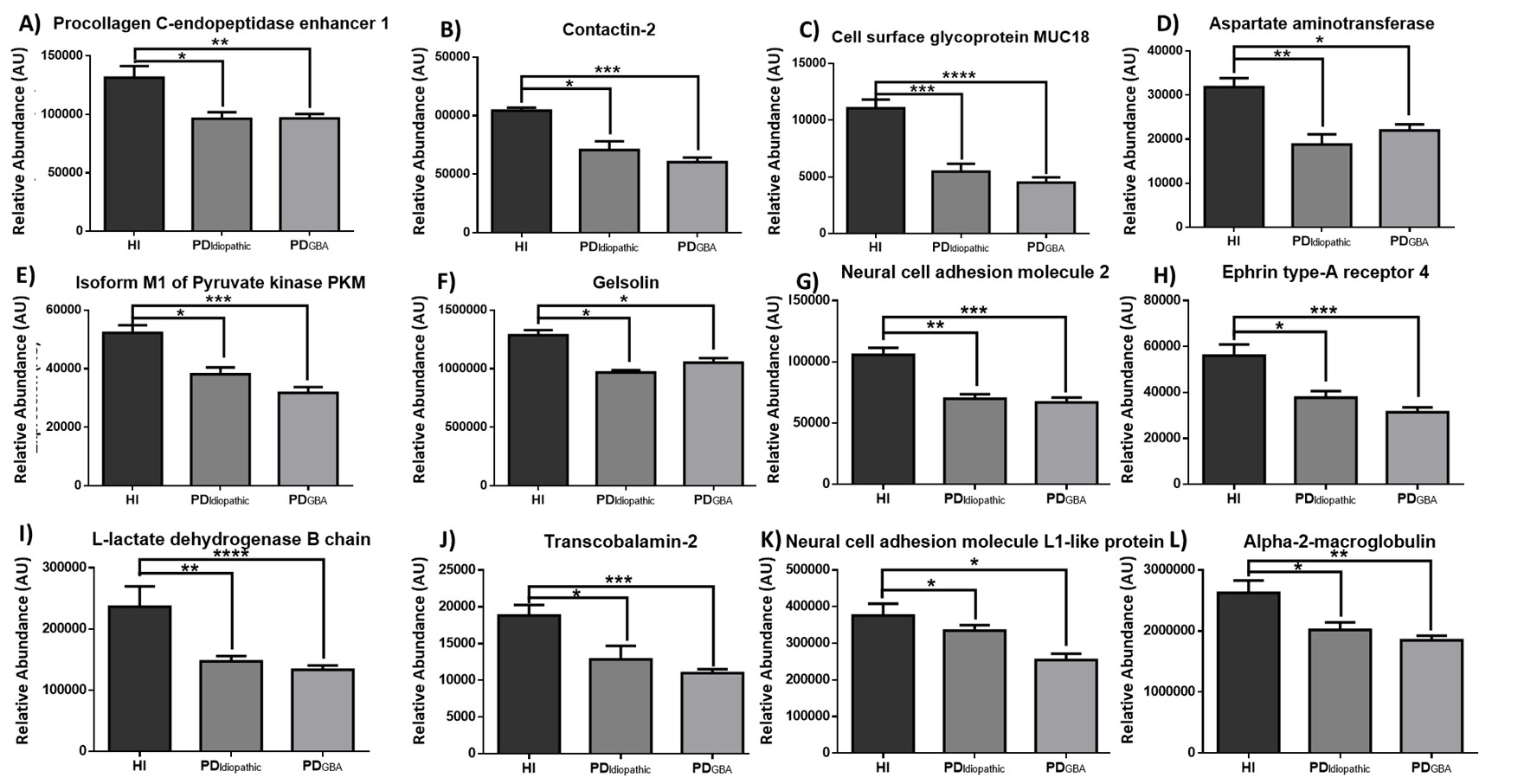

Supplement: Supplementary file 1 [file ijms-23-14166-s001.zip › Supplementary Figure 4.jpg]
